# Association Between COVID-19 Vaccine Doses and Neurosensory Symptoms Among COVID-19-Positive Patients: A Retrospective Cross-Sectional Study

**DOI:** 10.7759/cureus.116226

**Published:** 2026-09-14

**Authors:** Jaime Ricardo Sevillano Giraldez, Grace Thompson, Luis Escobar, Isaac Fernández Carmago, Luis Alberto Flores

**Affiliations:** 1 Epidemiology and Public Health, Región de Salud de San Miguelito, Ministerio de Salud Panamá, Panama City, PAN; 2 Public Health, University of South Florida, Tampa, USA; 3 Medical School, Universidad de Panamá, Panama City, PAN

**Keywords:** ageusia, anosmia, covid-19 vaccines, panama, pfizer-biontech, vaccine doses

## Abstract

Background: Ageusia and anosmia are common manifestations of COVID-19. This study examined whether their frequency among infected patients was associated with the recorded number of Pfizer-BioNTech vaccine doses; it was not designed to estimate causality or vaccine effectiveness.

Methods: We conducted a retrospective cross-sectional analysis of 5,348 COVID-19-positive patients over five years of age followed by the San Miguelito Health Region, Panama, from July 7, 2021, through November 2, 2022. Unvaccinated patients and vaccinated patients whose recorded doses were exclusively Pfizer-BioNTech were included. Separate logistic regression models estimated associations between vaccine dose category (zero, one, two, or three to four doses) and self-reported ageusia and anosmia, with two doses as the reference. A post hoc sensitivity analysis additionally adjusted for swab month.

Results: Ageusia was reported by 507 patients (9.48%), and anosmia by 584 patients (10.92%). In the primary adjusted models, compared with two doses, adjusted odds ratios (aORs) for zero, one, and three to four doses were 1.87 (95% confidence interval (CI) 1.48-2.38), 2.68 (95% CI 1.99-3.60), and 0.26 (95% CI 0.18-0.36), respectively, for ageusia, and 1.82 (95% CI 1.46-2.27), 2.53 (95% CI 1.91-3.35), and 0.18 (95% CI 0.12-0.25), respectively, for anosmia. After categorical adjustment for swab month, all six dose-category estimates were attenuated, and none remained statistically significant.

Conclusions: Primary models identified strong associations between recorded vaccine dose category and self-reported ageusia and anosmia among infected patients, but these associations did not persist after adjustment for swab month. The results are compatible with substantial confounding by epidemic period and should not be interpreted as causal effects or estimates of vaccine effectiveness.

## Introduction

Panama launched its national COVID-19 vaccination plan in January 2021 with phased priority for frontline health workers, older adults, people with chronic conditions, and other risk groups [1]. By October 11, 2021, the Ministry of Health reported that 75.5% of the vaccine-eligible population had completed the vaccination schedule and that 4,874,952 of 5,625,697 administered doses were Pfizer [2]. Pfizer-BioNTech (Pfizer Inc., New York, NY, USA) was therefore central to the national campaign and is the only manufacturer represented among vaccinated patients in the present analytical cohort.

Loss of smell (anosmia) and loss of taste (ageusia) are well-recognized manifestations of SARS-CoV-2 infection [3]. Although many patients recover, persistent dysfunction has been documented for up to one year after infection; in one Italian follow-up study, 31 of 178 participants (17.4%) reported persistent loss of both smell and taste [4].

Evidence on vaccination and chemosensory manifestations among infected patients remains heterogeneous. During Omicron-variant predominance, Song et al. found that receipt of three vaccine doses was associated with a lower prevalence of smell or taste alterations than fewer doses, but the observational design and close relationship between dose count and epidemic timing require cautious interpretation [5].

This study aimed to estimate the frequency of ageusia and anosmia among COVID-19-positive patients followed by the San Miguelito Health Region and to evaluate the association between the number of recorded COVID-19 vaccine doses and these symptoms. Because only infected patients were available and exposure and outcomes were assessed from the same follow-up record, the analysis does not establish temporality, causality, or vaccine effectiveness against infection or symptoms.

## Materials and methods

Study design and setting

We conducted a retrospective cross-sectional analysis of de-identified surveillance data from the San Miguelito Health Region (Región de Salud de San Miguelito (RSSM)), Panama. The verified analytical extract covered first follow-up calls from July 7, 2021, through November 2, 2022; corresponding swab dates ranged from July 5, 2021, through October 31, 2022. Physicians collected self-reported clinical and vaccination information during telephone follow-up calls. Because exact vaccination dates were not recorded, the timing of vaccination relative to SARS-CoV-2 infection could not be established.

Data source and access

The target population comprised individuals over five years of age who tested positive for COVID-19. The source population was drawn from the RSSM COVID-19 follow-up database, which contained 26,461 unverified operational records [6]. This institutional database is not publicly accessible and is not available online. Because it contains protected medical information, an anonymized dataset may be requested from RSSM/MINSA (Ministerio de Salud) only after all applicable Panamanian legal, regulatory, institutional, and ethics requirements governing access to medical information have been met. Access remains subject to institutional review and authorization and is not guaranteed.

The research team received a de-identified extract of 6,696 records. The extract was prepared operationally before delivery to the research team. It did not include a patient-level disposition log for the other 19,765 source records, so the reasons for their non-inclusion cannot be reconstructed. The research team did not individually screen or exclude those 19,765 records. Consequently, the 26,461 records are reported only as the unverified institutional source database, and selection bias introduced during pre-extraction processing cannot be excluded.

Participants

All records in the supplied extract represented a completed first follow-up call during home isolation. Records were eligible if the patient was over five years of age and was either unvaccinated or, if vaccinated, had received only Pfizer-BioNTech doses. We excluded 140 records for patients aged five years or younger, 1,198 records with a non-Pfizer vaccine or mixed-manufacturer schedule, and 10 records with an indeterminate vaccination category (vaccination marked as present but zero recorded doses and no manufacturer). The final analytical sample comprised 5,348 patients. The database contained no uninfected comparison group.

Variables and measurement

The main outcomes were self-reported ageusia and anosmia during the follow-up call. The primary exposure was the number of recorded vaccine doses, categorized as zero doses, one dose, two doses, and three to four doses. Two doses were the reference category. Zero doses represented unvaccinated patients; among patients with at least one dose, all recorded manufacturers were Pfizer-BioNTech. Exact vaccination dates, time since vaccination, prior SARS-CoV-2 infection, circulating variant, viral load, and sex were not recorded. Swab and call dates were available and were used to evaluate calendar-period confounding in a post hoc sensitivity analysis.

Age was analyzed continuously and in four nominal groups: 6-17, 18-29, 30-62, and 63 years or older. In categorical models, age group was dummy-coded with 30-62 years as the reference; it was not treated as a one-step ordinal score. Binary variables used 0=No as the reference and 1=Yes. The number of other symptoms was the sum of 12 recorded symptoms excluding ageusia and anosmia. The number of chronic conditions was the sum of hypertension, diabetes, cancer, chronic kidney disease, obesity, tuberculosis, chronic obstructive pulmonary disease (COPD), asthma, and immunosuppression. Any previous hospitalization was analyzed separately.

Data cleaning and quality control

Cleaning followed the RACE framework (integrity, consistency, completeness, and accuracy). Variable names and vaccine manufacturer labels were standardized. A total of 14 swab dates had been imported as May 1 or June 1, 2022, although their January 7-8, 2022 call dates made that sequence impossible; reversing the imported day and month yielded January 5-6, 2022 and placed each call zero to two days after the swab. These deterministic corrections were restricted to the 14 identified records, whose anonymized identifiers and original and corrected values are retained in the reproducible cleaning script; the source workbook was not overwritten. Structural manufacturer fields for unreceived doses were labeled not applicable. Any exact zero in the original previous-hospitalization field was coded "No"; any nonzero response or description of a prior admission was coded "Yes". No values were imputed, and all variables used in the final analyses were complete after the prespecified exclusions. Variables not collected were treated as unavailable rather than imputed. A total of 29 pairs of identical analytical profiles were retained because every record had a distinct anonymized identifier. Extreme symptom and comorbidity counts were clinically plausible component sums and were retained for the primary analysis.

Statistical methods

Continuous variables were summarized using means and standard deviations (SD), and categorical variables were reported as counts and column percentages. Additionally, a separate univariable logistic regression model was fitted for every predictor and for each outcome. These analyses were exploratory; no multiplicity adjustment was applied, and individual p-values should not be interpreted as confirmatory evidence. No additional predictor was included in those models. Age group was modeled as a nominal factor with 30-62 years as the reference. Vaccine category used two doses as the reference. Binary predictors used "No" as the reference. Continuous age was modeled per one-year increase, and symptom and comorbidity counts were modeled per one-unit increase. All models used 5,348 patients, including 507 ageusia events and 584 anosmia events. Results are presented as odds ratios (ORs), 95% confidence intervals (CIs), and separate two-sided Wald p-values for ageusia and anosmia.

Tuberculosis and COPD were analyzed separately because no exposed patient experienced either outcome. Crude risk ratios (RRs) were calculated as the risk among exposed patients divided by the risk among unexposed patients. For each zero-event exposure, the two-sided 95% CI was obtained by profile likelihood under independent binomial sampling, with the lower limit at the boundary of zero. Two-sided Fisher exact tests supplied the outcome-specific p-values.

Three logistic models estimated the association of vaccine category with each outcome. Model I was unadjusted. Model II adjusted for age, number of other symptoms, and number of chronic conditions. Model III additionally adjusted for headache, fever, runny nose, and difficulty breathing [3,7,8]. Because the relationship of age with the logit was nonlinear, age was modeled with a natural cubic spline with three degrees of freedom. The other-symptom count was also modeled with a three-degree-of-freedom natural spline; the chronic-condition count was retained as a linear term after testing a quadratic extension. Model fit, convergence, events per parameter, generalized variance inflation factors, discrimination, fitted probabilities, and influence were examined.

Three sensitivity analyses were performed. First, Model III was repeated after excluding 121 records flagged by the Tukey rule for unusually high symptom or comorbidity counts. Second, adjusted prevalence ratios were estimated with Poisson regression and a robust HC0 sandwich variance because outcome prevalence was near 10%. Third, after peer review, Model III was repeated with categorical swab month added to directly evaluate calendar-period confounding. Statistical tests were two-sided with α=0.05. Sensitivity analyses assess stability under specified models but cannot eliminate unmeasured confounding. All cleaning, analyses, tables, and figures were generated using R version 4.6.1 (R Foundation for Statistical Computing, Vienna, Austria).

Use of artificial intelligence-assisted tools

During manuscript revision, the authors used OpenAI Codex (OpenAI, San Francisco, CA, USA) to assist with data-cleaning verification, generation and verification of reproducible R code, table restructuring, reference-link verification, and language editing. The submitted figures are deterministic numerical displays generated from the study data in R; no generative image model was used to create or modify them. The authors made all scientific decisions, selected the analyses, interpreted the results, checked all numerical and textual outputs against the source data, and took full responsibility for the final content. The artificial intelligence tool was not listed as an author and did not make autonomous scientific or publication decisions.

Ethical considerations

The Bioethics Committee of the Universidad Metropolitana de Educación, Ciencia y Tecnología (UMECIT) approved the protocol (CBIU-2025-5-0011) on May 30, 2025. Permission to access the database was obtained from the medical chief of the San Miguelito Health Region, the national chief of epidemiology, and the national director of health. The study used de-identified secondary surveillance data, and informed consent for this secondary analysis was waived as documented by the ethics approval.

## Results

Participants

The institutional RSSM database contained 26,461 unverified operational records. The research team received a de-identified extract of 6,696 records; the extract did not contain a patient-level disposition log linking the operational source to the analytical file. After excluding 140 records for age five years or younger, 1,198 records with non-Pfizer or mixed vaccination, and 10 records with indeterminate vaccination status, 5,348 records remained. Figure 1 summarizes the derivation of the analytical cohort.

**Figure 1:**
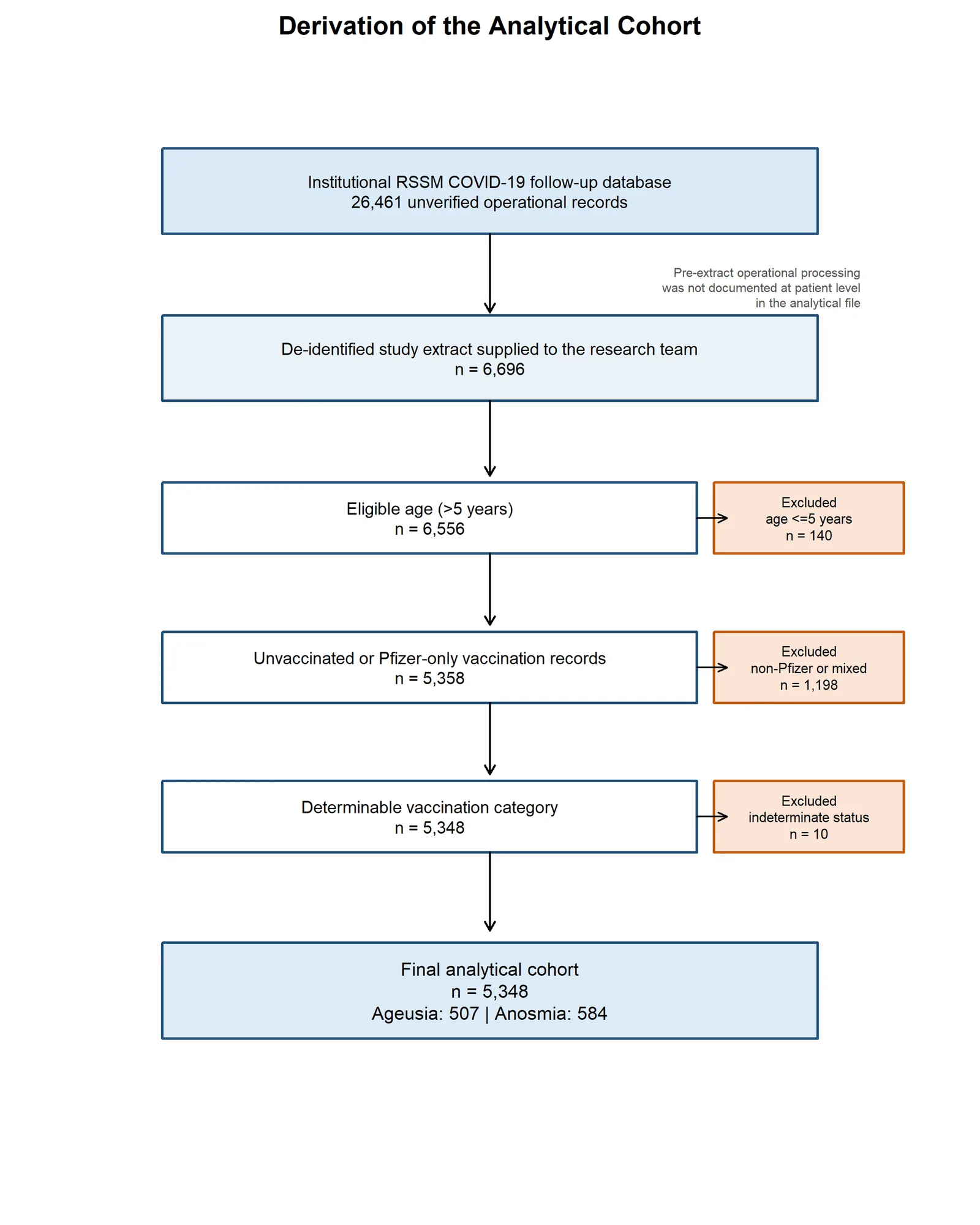
Derivation of the Analytical Cohort The San Miguelito Health Region institutional database contained 26,461 unverified operational records. The research team received a de-identified extract of 6,696 records; no patient-level disposition log was available for the other 19,765 source records. Exclusions from the supplied extract were age five years or younger (n=140), non-Pfizer or mixed vaccination (n=1,198), and indeterminate vaccination category (n=10), leaving 5,348 patients.

Descriptive data

The mean age was 47.36±21.31 years. Mean age differed substantially by vaccine category: 33.32±19.34 years for zero doses, 33.92±15.78 for one dose, 45.10±20.65 for two doses, and 58.54±17.10 for three to four doses, demonstrating strong measured confounding by age. The cohort included 1,097 patients with zero doses (20.51%), 362 with one dose (6.77%), 1,728 with two doses (32.31%), and 2,161 with three to four doses (40.41%). At least one symptom was reported by 4,272 patients (79.88%). The most frequent recorded symptoms were cough in 2,499 patients (46.73%), runny nose in 2,436 patients (45.55%), sore throat in 1,410 patients (26.36%), and headache in 922 patients (17.24%). At least one chronic condition was present in 1,649 patients (30.83%); hypertension was recorded in 1,254 patients (23.45%) and diabetes in 464 patients (8.68%). Table 1 presents participant characteristics by vaccine dose category.

**Table 1:** Participant Characteristics by COVID-19 Vaccine Dose Category (N=5,348) Values are n (column %) unless otherwise stated. Age is shown as mean±SD. Zero doses denotes unvaccinated patients. Among vaccinated patients, all recorded doses were Pfizer-BioNTech. COPD, chronic obstructive pulmonary disease; SD, standard deviation.

Characteristic	Level	Overall	0 doses	1 dose	2 doses	3-4 doses
Participants	n	5348	1097	362	1728	2161
Age, years	Mean±SD	47.36±21.31	33.32±19.34	33.92±15.78	45.10±20.65	58.54±17.10
Age group, n (%)	6-17	556 (10.40%)	273 (24.89%)	50 (13.81%)	188 (10.88%)	45 (2.08%)
Age group, n (%)	18-29	876 (16.38%)	253 (23.06%)	124 (34.25%)	332 (19.21%)	167 (7.73%)
Age group, n (%)	30-62	2,378 (44.47%)	483 (44.03%)	178 (49.17%)	824 (47.69%)	893 (41.32%)
Age group, n (%)	63+	1,538 (28.76%)	88 (8.02%)	10 (2.76%)	384 (22.22%)	1,056 (48.87%)
Any symptom	Yes, n (%)	4,272 (79.88%)	822 (74.93%)	306 (84.53%)	1,394 (80.67%)	1,750 (80.98%)
Headache	Yes, n (%)	922 (17.24%)	222 (20.24%)	110 (30.39%)	295 (17.07%)	295 (13.65%)
Fever	Yes, n (%)	631 (11.80%)	171 (15.59%)	62 (17.13%)	179 (10.36%)	219 (10.13%)
Runny nose	Yes, n (%)	2,436 (45.55%)	379 (34.55%)	182 (50.28%)	831 (48.09%)	1,044 (48.31%)
Sore throat	Yes, n (%)	1,410 (26.36%)	203 (18.51%)	81 (22.38%)	453 (26.22%)	673 (31.14%)
Cough	Yes, n (%)	2,499 (46.73%)	421 (38.38%)	164 (45.30%)	796 (46.06%)	1,118 (51.74%)
Vomiting	Yes, n (%)	34 (0.64%)	12 (1.09%)	7 (1.93%)	4 (0.23%)	11 (0.51%)
Diarrhea	Yes, n (%)	125 (2.34%)	43 (3.92%)	9 (2.49%)	39 (2.26%)	34 (1.57%)
Dyspnea	Yes, n (%)	22 (0.41%)	4 (0.36%)	1 (0.28%)	7 (0.41%)	10 (0.46%)
Altered alertness	Yes, n (%)	7 (0.13%)	4 (0.36%)	0 (0.00%)	1 (0.06%)	2 (0.09%)
Chest pain	Yes, n (%)	118 (2.21%)	42 (3.83%)	8 (2.21%)	40 (2.31%)	28 (1.30%)
Mood changes	Yes, n (%)	11 (0.21%)	5 (0.46%)	0 (0.00%)	3 (0.17%)	3 (0.14%)
Difficulty breathing	Yes, n (%)	52 (0.97%)	20 (1.82%)	5 (1.38%)	9 (0.52%)	18 (0.83%)
Ageusia	Yes, n (%)	507 (9.48%)	192 (17.50%)	99 (27.35%)	171 (9.90%)	45 (2.08%)
Anosmia	Yes, n (%)	584 (10.92%)	224 (20.42%)	110 (30.39%)	209 (12.09%)	41 (1.90%)
Any chronic condition	Yes, n (%)	1,649 (30.83%)	68 (6.20%)	11 (3.04%)	440 (25.46%)	1,130 (52.29%)
Hypertension	Yes, n (%)	1,254 (23.45%)	47 (4.28%)	4 (1.10%)	297 (17.19%)	906 (41.93%)
Diabetes	Yes, n (%)	464 (8.68%)	22 (2.01%)	2 (0.55%)	102 (5.90%)	338 (15.64%)
Cancer	Yes, n (%)	95 (1.78%)	1 (0.09%)	0 (0.00%)	26 (1.50%)	68 (3.15%)
Chronic kidney disease	Yes, n (%)	14 (0.26%)	0 (0.00%)	0 (0.00%)	2 (0.12%)	12 (0.56%)
Obesity	Yes, n (%)	71 (1.33%)	3 (0.27%)	1 (0.28%)	23 (1.33%)	44 (2.04%)
Tuberculosis	Yes, n (%)	5 (0.09%)	0 (0.00%)	1 (0.28%)	1 (0.06%)	3 (0.14%)
COPD	Yes, n (%)	11 (0.21%)	0 (0.00%)	0 (0.00%)	4 (0.23%)	7 (0.32%)
Asthma	Yes, n (%)	215 (4.02%)	17 (1.55%)	6 (1.66%)	84 (4.86%)	108 (5.00%)
Immunosuppression	Yes, n (%)	17 (0.32%)	1 (0.09%)	0 (0.00%)	4 (0.23%)	12 (0.56%)
Any previous hospitalization	Yes, n (%)	358 (6.69%)	20 (1.82%)	4 (1.10%)	97 (5.61%)	237 (10.97%)

Outcome data

Ageusia occurred in 507 patients (9.48%), and anosmia occurred in 584 patients (10.92%). Ageusia was reported by 192 patients with zero doses (17.50%), 99 with one dose (27.35%), 171 with two doses (9.90%), and 45 with three to four doses (2.08%). Anosmia was reported by 224 patients with zero doses (20.42%), 110 with one dose (30.39%), 209 with two doses (12.09%), and 41 with three to four doses (1.90%).

Univariable analysis

Separate univariable logistic regression models were fitted for every predictor and outcome as exploratory analyses without multiplicity adjustment. Relative to two doses, zero and one dose were associated with higher odds of both outcomes, whereas three to four doses were associated with lower odds. Age, age group, the number of other symptoms, several individual symptoms, the number of chronic conditions, hypertension, diabetes, and previous hospitalization were also associated with one or both outcomes. Given the number of comparisons and the potential for confounding, these individual findings are descriptive and hypothesis-generating. Table 2 reports the complete univariable results with reference categories and separate p-value columns.

**Table 2:** Separate Univariable Logistic Regression Models for Ageusia and Anosmia (N=5,348) Each row derives from a separate univariable logistic regression for each outcome. N=5,348, including 507 ageusia events and 584 anosmia events. Two doses are the vaccination reference. Age group is nominal and dummy-coded with 30-62 years as reference. Binary variables use No as reference. OR, odds ratio; CI, confidence interval.

Variable	Comparison or unit	N	Ageusia OR (95% CI)	Ageusia p	Anosmia OR (95% CI)	Anosmia p
Vaccination category	2 doses (reference)	5348	Reference	-	Reference	-
Vaccination category	0 doses vs 2 doses	5348	1.93 (1.55-2.41)	<0.001	1.86 (1.52-2.29)	<0.001
Vaccination category	1 dose vs 2 doses	5348	3.43 (2.59-4.53)	<0.001	3.17 (2.43-4.14)	<0.001
Vaccination category	3-4 doses vs 2 doses	5348	0.19 (0.14-0.27)	<0.001	0.14 (0.10-0.20)	<0.001
Age	Per 1-year increase	5348	0.97 (0.97-0.98)	<0.001	0.97 (0.97-0.98)	<0.001
Age group	30-62 years (reference)	5348	Reference	-	Reference	-
Age group	6-17 vs 30-62 years	5348	0.91 (0.66-1.25)	0.543	0.67 (0.49-0.93)	0.016
Age group	18-29 vs 30-62 years	5348	2.40 (1.94-2.97)	<0.001	2.18 (1.79-2.67)	<0.001
Age group	63+ vs 30-62 years	5348	0.25 (0.18-0.35)	<0.001	0.23 (0.17-0.32)	<0.001
Number of other symptoms	Per 1-symptom increase; excludes ageusia and anosmia	5348	1.34 (1.26-1.42)	<0.001	1.26 (1.19-1.34)	<0.001
Headache	Yes vs No (reference)	5348	2.83 (2.32-3.45)	<0.001	2.37 (1.95-2.87)	<0.001
Fever	Yes vs No (reference)	5348	1.88 (1.48-2.39)	<0.001	1.65 (1.30-2.08)	<0.001
Runny nose	Yes vs No (reference)	5348	1.89 (1.57-2.27)	<0.001	1.68 (1.41-2.00)	<0.001
Sore throat	Yes vs No (reference)	5348	1.14 (0.93-1.40)	0.192	0.98 (0.81-1.19)	0.844
Cough	Yes vs No (reference)	5348	1.28 (1.06-1.53)	0.009	1.17 (0.98-1.39)	0.077
Vomiting	Yes vs No (reference)	5348	4.65 (2.25-9.59)	<0.001	2.97 (1.38-6.39)	0.005
Diarrhea	Yes vs No (reference)	5348	2.59 (1.66-4.03)	<0.001	2.31 (1.49-3.57)	<0.001
Dyspnea	Yes vs No (reference)	5348	3.61 (1.41-9.27)	0.008	3.08 (1.20-7.90)	0.019
Altered alertness	Yes vs No (reference)	5348	7.20 (1.61-32.25)	0.01	6.14 (1.37-27.52)	0.018
Chest pain	Yes vs No (reference)	5348	3.90 (2.58-5.89)	<0.001	3.60 (2.40-5.39)	<0.001
Mood changes	Yes vs No (reference)	5348	2.13 (0.46-9.87)	0.335	4.69 (1.37-16.06)	0.014
Difficulty breathing	Yes vs No (reference)	5348	2.30 (1.15-4.61)	0.019	4.42 (2.48-7.89)	<0.001
Number of chronic conditions	Per 1-condition increase	5348	0.39 (0.31-0.48)	<0.001	0.38 (0.31-0.47)	<0.001
Hypertension	Yes vs No (reference)	5348	0.20 (0.14-0.29)	<0.001	0.20 (0.14-0.28)	<0.001
Diabetes	Yes vs No (reference)	5348	0.28 (0.16-0.48)	<0.001	0.24 (0.14-0.40)	<0.001
Cancer	Yes vs No (reference)	5348	0.53 (0.21-1.30)	0.164	0.55 (0.24-1.25)	0.153
Chronic kidney disease	Yes vs No (reference)	5348	0.73 (0.10-5.62)	0.766	0.63 (0.08-4.79)	0.653
Obesity	Yes vs No (reference)	5348	0.72 (0.29-1.80)	0.482	0.89 (0.41-1.95)	0.773
Asthma	Yes vs No (reference)	5348	0.87 (0.53-1.42)	0.572	0.83 (0.52-1.33)	0.438
Immunosuppression	Yes vs No (reference)	5348	0.60 (0.08-4.50)	0.616	1.09 (0.25-4.77)	0.911
Any previous hospitalization	Yes vs No (reference)	5348	0.40 (0.24-0.68)	<0.001	0.39 (0.24-0.64)	<0.001

Sparse-Exposure Analysis

There were five patients with tuberculosis and 11 patients with COPD. None reported ageusia or anosmia. For tuberculosis, the crude RR was 0.00 (95% CI 0.00-3.37; p=1.000) for ageusia and 0.00 (95% CI 0.00-2.92; p=1.000) for anosmia. For COPD, the crude RR was 0.00 (95% CI 0.00-1.69; p=0.614) for ageusia and 0.00 (95% CI 0.00-1.47; p=0.622) for anosmia. Because the exposed groups were extremely small and CIs were wide, these estimates are reported only for completeness and do not support reliable clinical inference. Table 3 presents the outcome-specific results.

**Table 3:** Crude Risk Ratios for Tuberculosis and COPD With Zero Outcome Events (N=5,348) RR=risk in exposed/risk in unexposed. No tuberculosis-exposed or COPD-exposed patient had ageusia or anosmia. Two-sided 95% profile-likelihood CIs were calculated under independent binomial sampling; the lower limit lies at zero. p-values are two-sided Fisher exact tests. COPD, chronic obstructive pulmonary disease; RR, risk ratio; CI, confidence interval.

Exposure	Exposed, n	Ageusia events, n	Ageusia RR (95% CI)	Ageusia p	Anosmia events, n	Anosmia RR (95% CI)	Anosmia p
Tuberculosis	5	0	0.00 (0.00-3.37)	1	0	0.00 (0.00-2.92)	1
COPD	11	0	0.00 (0.00-1.69)	0.614	0	0.00 (0.00-1.47)	0.622

Multivariable analysis

In the prespecified Model III, compared with two doses, zero doses were associated with ageusia (aOR 1.87, 95% CI 1.48-2.38; p<0.001) and anosmia (aOR 1.82, 95% CI 1.46-2.27; p<0.001). One dose was associated with ageusia (aOR 2.68, 95% CI 1.99-3.60; p<0.001) and anosmia (aOR 2.53, 95% CI 1.91-3.35; p<0.001). Three to four doses were associated with lower odds of ageusia (aOR 0.26, 95% CI 0.18-0.36; p<0.001) and anosmia (aOR 0.18, 95% CI 0.12-0.25; p<0.001). Crude and adjusted estimates are shown in Table 4. Figure 2 displays the primary Model III estimates, which did not include swab month.

**Table 4:** Crude and Adjusted Associations Between Vaccine Dose Category and Neurosensory Outcomes (N=5,348) Reference category: two doses. Model I is unadjusted. Model II adjusts for age and number of other symptoms using three-degree-of-freedom natural cubic splines and for number of chronic conditions as a linear term. Model III additionally adjusts for headache, fever, runny nose, and difficulty breathing. OR, odds ratio; CI, confidence interval.

Outcome	Vaccine dose category	N	Events, n (%)	Model I OR (95% CI)	Model I p	Model II OR (95% CI)	Model II p	Model III OR (95% CI)	Model III p
Ageusia	0 doses	1097	192 (17.50%)	1.93 (1.55-2.41)	<0.001	1.78 (1.41-2.25)	<0.001	1.87 (1.48-2.38)	<0.001
Ageusia	1 dose	362	99 (27.35%)	3.43 (2.59-4.53)	<0.001	2.68 (2.00-3.59)	<0.001	2.68 (1.99-3.60)	<0.001
Ageusia	2 doses	1728	171 (9.90%)	Reference	-	Reference	-	Reference	-
Ageusia	3-4 doses	2161	45 (2.08%)	0.19 (0.14-0.27)	<0.001	0.25 (0.18-0.35)	<0.001	0.26 (0.18-0.36)	<0.001
Anosmia	0 doses	1097	224 (20.42%)	1.86 (1.52-2.29)	<0.001	1.74 (1.40-2.16)	<0.001	1.82 (1.46-2.27)	<0.001
Anosmia	1 dose	362	110 (30.39%)	3.17 (2.43-4.14)	<0.001	2.52 (1.90-3.33)	<0.001	2.53 (1.91-3.35)	<0.001
Anosmia	2 doses	1728	209 (12.09%)	Reference	-	Reference	-	Reference	-
Anosmia	3-4 doses	2161	41 (1.90%)	0.14 (0.10-0.20)	<0.001	0.18 (0.12-0.25)	<0.001	0.18 (0.12-0.25)	<0.001

**Figure 2:**
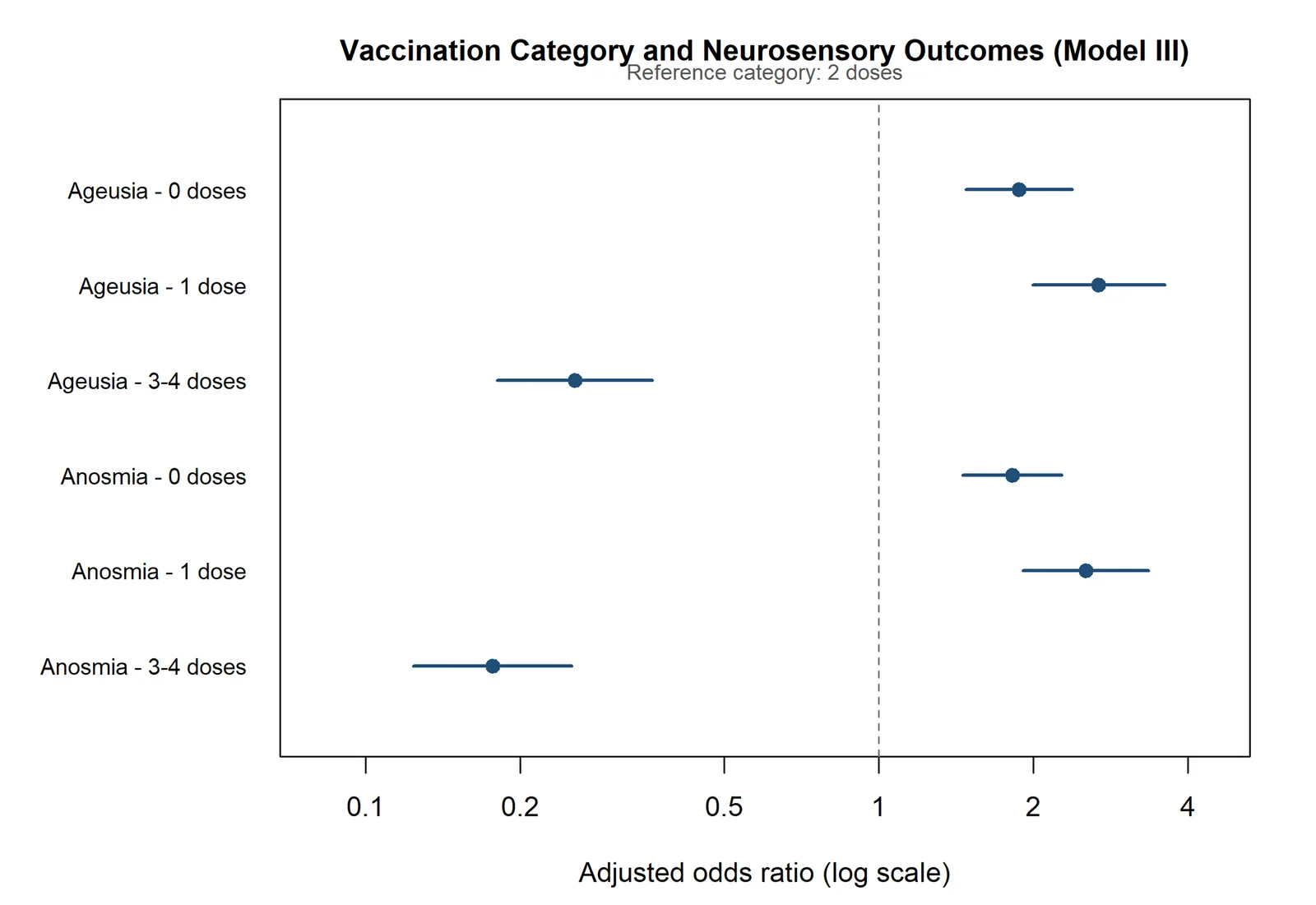
Primary Model III Adjusted Odds Ratios for Ageusia and Anosmia by Vaccine Dose Category Points show adjusted odds ratios and lines show 95% confidence intervals from the primary Model III; two doses are the reference. Model III adjusted for age and other-symptom count using natural cubic splines with three degrees of freedom, chronic-condition count, headache, fever, runny nose, and difficulty breathing. The figure does not include calendar-month adjustment; post hoc calendar-adjusted estimates are reported in Table 5.

Model checks and sensitivity analyses

Both primary Model III fits converged with finite coefficients. Events per parameter were 33.8 for ageusia and 38.9 for anosmia, and the maximum adjusted generalized variance inflation factor was 1.43. Exclusion of 121 Tukey-flagged records left 5,227 patients and did not change the direction or statistical significance of the primary estimates; robust Poisson models also gave the same directional interpretation. However, adding categorical swab month attenuated all six vaccine-category estimates, and none remained statistically significant: for ageusia, aORs were 0.93 (95% CI 0.68-1.28; p=0.649), 1.14 (95% CI 0.79-1.65; p=0.491), and 0.77 (95% CI 0.49-1.23; p=0.279) for zero, one, and three to four doses, respectively; for anosmia, the corresponding aORs were 0.78 (95% CI 0.57-1.05; p=0.098), 0.97 (95% CI 0.68-1.38; p=0.861), and 0.65 (95% CI 0.41-1.04; p=0.072). Table 5 reports the post hoc calendar-month sensitivity analysis.

**Table 5:** Post Hoc Calendar-Month Sensitivity Analysis of Vaccine Dose Category and Neurosensory Outcomes (N=5,348) Two doses are the reference category. The primary Model III adjusted for age and number of other symptoms using three-degree-of-freedom natural splines, chronic-condition count, headache, fever, runny nose, and difficulty breathing. The post hoc model additionally included categorical swab month. None of the six calendar-adjusted estimates was statistically significant at α=0.05. aOR, adjusted odds ratio; CI, confidence interval.

Outcome	Dose category	Primary Model III aOR (95% CI)	Primary p-value	Model III plus swab month aOR (95% CI)	Calendar-adjusted p-value
Ageusia	0 doses	1.87 (1.48-2.38)	<0.001	0.93 (0.68-1.28)	0.649
Ageusia	1 dose	2.68 (1.99-3.60)	<0.001	1.14 (0.79-1.65)	0.491
Ageusia	3-4 doses	0.26 (0.18-0.36)	<0.001	0.77 (0.49-1.23)	0.279
Anosmia	0 doses	1.82 (1.46-2.27)	<0.001	0.78 (0.57-1.05)	0.098
Anosmia	1 dose	2.53 (1.91-3.35)	<0.001	0.97 (0.68-1.38)	0.861
Anosmia	3-4 doses	0.18 (0.12-0.25)	<0.001	0.65 (0.41-1.04)	0.072

## Discussion

Key findings

In the primary analysis of 5,348 COVID-19-positive patients, recorded vaccine dose category was strongly associated with self-reported ageusia and anosmia after adjustment for measured clinical covariates. Outlier-exclusion and robust prevalence-ratio analyses preserved the direction of those primary estimates. In contrast, the associations were markedly attenuated and no longer statistically significant after categorical adjustment for swab month, indicating that epidemic period was an important confounder.

Comparison with other studies

The direction of the unadjusted and primary adjusted estimates was similar to a Chinese cross-sectional study in which three-dose vaccination was associated with a lower prevalence of smell or taste alterations during Omicron-predominant periods [5]. A nationwide Danish study also associated booster vaccination with fewer post-acute symptoms after Omicron infection than two doses [9], while another Chinese study found that vaccination-related patterns varied by symptom and time since vaccination [10]. Direct comparison is limited by differences in populations, outcome timing, vaccine products, epidemic periods, and covariate availability. The loss of statistical significance after swab-month adjustment in the present study reinforces the need to model calendar time and variant rather than interpret dose count in isolation.

A Brazilian observational preprint reported product-specific symptom associations that did not uniformly favor vaccinated groups [11]. Differences in sampling, vaccine products, weighting, comparison groups, and epidemic periods may explain the differing estimates. The present study is restricted to infected patients and compares four recorded dose categories; it cannot estimate protection against infection or the causal effect of vaccination on symptoms.

Vaccination does not eliminate neurosensory symptoms in every breakthrough infection. Caponio et al. described ageusia after symptomatic SARS-CoV-2 infection following two BNT162b2 doses [12], and Sagar et al. observed substantial olfactory and gustatory dysfunction in a small prospective cohort of breakthrough infections [13]. In the present cohort, 171 patients in the two-dose group (9.90%) and 45 in the three-to-four-dose group (2.08%) reported ageusia. Huhn et al. reported relationships between antibody responses, ACE2 neutralization, and anosmia or ageusia, providing biological context without establishing a dose effect [14]. A systematic review also identified rare reports of anosmia or ageusia after COVID-19 vaccination [15]; those post-vaccination events are clinically distinct from symptoms recorded after confirmed infection in this study.

Limitations

Several limitations are important. First, the institutional source contained 26,461 unverified operational records, whereas the research team received a 6,696-record de-identified extract without a patient-level disposition log for the other 19,765 records. Selection before delivery cannot be reconstructed and may have produced a nonrepresentative analytical sample. Second, the cross-sectional design does not establish whether vaccination preceded the relevant biological window for symptom development and cannot establish causality. Third, vaccine dose category was strongly related to age and calendar time. Exact vaccination dates, time since vaccination, prior infections, circulating variant, viral load, sex, and socioeconomic variables were unavailable. Measured adjustment cannot correct for these unmeasured or incompletely measured factors. The disappearance of all six associations after swab-month adjustment shows that epidemic-period confounding could materially account for the primary estimates.

Fourth, symptoms and vaccination history were self-reported by telephone, creating potential misclassification and measurement bias. Fifth, only unvaccinated patients and patients whose recorded doses were exclusively Pfizer-BioNTech were included, limiting generalizability to other vaccine platforms. Sixth, the study was conducted in a single urban health region and included only COVID-19-positive patients, so the estimates do not measure vaccine effectiveness against infection and may not apply to other settings. Seventh, Table 2 involved multiple exploratory comparisons without multiplicity adjustment. Eighth, odds ratios can differ from prevalence ratios when outcomes are not rare; robust prevalence-ratio sensitivity analyses preserved the primary direction but did not address unmeasured confounding. Finally, tuberculosis and COPD were uncommon, so their zero-event estimates are unreliable and should not be interpreted as evidence of protection.

Interpretation and generalizability

The results are most directly applicable to COVID-19-positive patients over five years of age in an urban Panamanian health-region follow-up system during July 2021-November 2022. The primary findings describe associations in the supplied analytical extract, not vaccine effects. Because the estimates were strongly attenuated after swab-month adjustment, they are compatible with differences among dose groups across epidemic periods rather than a direct dose-response relationship. Longitudinal analyses with documented sampling, exact vaccination dates, variant information, prior infection history, and prospectively measured outcomes are needed.

## Conclusions

Among COVID-19-positive patients in the RSSM follow-up cohort, primary models identified strong associations between recorded Pfizer-BioNTech dose category and self-reported ageusia and anosmia. Those associations remained directionally consistent in outlier-exclusion and robust prevalence-ratio analyses but were attenuated and no longer statistically significant after adjustment for swab month.

These findings should not be interpreted as causal effects or estimates of vaccine effectiveness. The calendar-month sensitivity analysis indicates that epidemic-period confounding is substantial, while selection before extract delivery, circulating variant, vaccination timing, prior infection, and other unmeasured factors remain unresolved. Confirmation requires longitudinal studies with a documented sampling frame and time-resolved exposure and infection data.
